# Enabling network inference methods to handle missing data and outliers

**DOI:** 10.1186/s12859-015-0717-7

**Published:** 2015-09-03

**Authors:** Abel Folch-Fortuny, Alejandro F. Villaverde, Alberto Ferrer, Julio R. Banga

**Affiliations:** 1 Departamento de Estadística e Investigación Operativa Aplicadas y Calidad, Universitat Politècnica de València, Camino de Vera s/n, Valencia, 46022 Spain; 2 BioProcess Engineering Group, IIM-CSIC, Eduardo Cabello 6, Vigo, 36208 Spain; 3 Centre of Biological Engineering, Universidade do Minho, Campus de Gualtar, Braga, 4710–057 Portugal; 4 Department of Systems and Control Engineering, Universidade de Vigo, Rua Maxwell, Vigo, 36310 Spain

**Keywords:** Network inference, Missing data, Outlier detection, Projection to latent structures, Trimmed scores regression, Information theory, Mutual information

## Abstract

**Background:**

The inference of complex networks from data is a challenging problem in biological sciences, as well as in a wide range of disciplines such as chemistry, technology, economics, or sociology. The quantity and quality of the data greatly affect the results. While many methodologies have been developed for this task, they seldom take into account issues such as missing data or outlier detection and correction, which need to be properly addressed before network inference.

**Results:**

Here we present an approach to (i) handle missing data and (ii) detect and correct outliers based on multivariate projection to latent structures. The method, called trimmed scores regression (TSR), enables network inference methods to analyse incomplete datasets by imputing the missing values coherently with the latent data structure. Furthermore, it substitutes the faulty values in a dataset by proper estimations. We provide an implementation of this approach, and show how it can be integrated with any network inference method as a preliminary data curation step. This functionality is demonstrated with a state of the art network inference method based on mutual information distance and entropy reduction, MIDER.

**Conclusion:**

The methodology presented here enables network inference methods to analyse a large number of incomplete and faulty datasets that could not be reliably analysed so far. Our comparative studies show the superiority of TSR over other missing data approaches used by practitioners. Furthermore, the method allows for outlier detection and correction.

**Electronic supplementary material:**

The online version of this article (doi:10.1186/s12859-015-0717-7) contains supplementary material, which is available to authorized users.

## Background

The problem of inferring complex networks is frequently addressed in several research areas [1, 2] such as social sciences (networks between academics, labor relations), engineering (electric power grid), chemistry (chemical reaction networks) or biology (ecological or cellular networks). In the context of cellular networks the individuals (network nodes) are biochemical entities such as genes, proteins, or metabolites. Many different approaches have been applied to date in this area, none of which can be singled out as the best one for all problems. Comparisons often find large discrepancies between the predictions of different algorithms, making network inference an open problem in bioinformatics research [3–7].

Network reconstruction usually begins with collecting data from each individual that participates in the network. Then, based on the relationships detected among them, links between the individuals are established. When no prior knowledge is introduced, the reconstruction of complex networks depends solely on the available data. In many cases, the data collection represents a challenge itself when experimental measurements are involved. Network inference methods rely on estimating quantities such as correlation or mutual information, whose calculation requires simultaneous measurements of several variables. When the data collection in a time point fails for a particular variable, resulting in an unmeasured value, the scientist has to decide whether to discard the information regarding the entire experiment at this time point or to impute an appropriate value. This problem is also faced in process monitoring, for example when sensors fail, when a particular measurement is outside the range of the instrument, or when a failure is produced between the control system and the instrumentation [8, 9].

The problem of missing data has been widely studied using projection to latent structure methods such as principal component analysis (PCA) [10]. Several methods have been developed using PCA in their cores, like the iterative algorithm (IA) [11], the NIPALS algorithm [12], and regression-based methods like known data regression (KDR) and trimmed scores regression (TSR) [13], of which the latter has been shown to be one of the best performers [14]. These methods exploit the existing correlations between variables to impute the missing values coherently with the latent structure of the dataset. Other methods commonly applied to impute missing data are based on clustering algorithms, like the *k*-nearest neighbor algorithm and its variants [15].

Projection to latent structure methods, especially PCA, can also be used for outlier detection [16]. Multivariate statistical process control allows the detection of abnormal situations during process monitoring [17]. Latent structure-based methods allow monitoring a large number of process variables, reducing the dimensionality of the original space to a few latent variables and statistics that are easy to control using charts. These methods are able to detect not only univariate outliers (*i.e.* values outside the usual range of a particular variable) but also changes in the latent structure (*i.e.* correlation structure) of the original variables, which classical univariate control charts usually miss [18]. Latent structure-based monitoring methods can be easily extrapolated to network inference. In this context, the outliers would be abnormal behaviors for certain individuals of the original dataset, or odd measurements for concentrations in time points during an experiment.

**Fig. 1:**
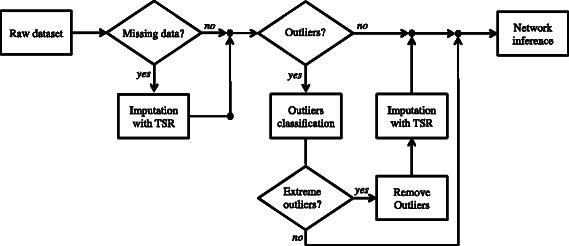
Missing data and outlier detection and correction modules. Initially, if there are missing values in the raw dataset, trimmed scores regression (TSR) method is used to impute the missing data. Then, if the dataset has extreme outliers, first a missing value is generated for the faulty observation, and secondly the observation is reconstructed using again TSR

In this work we provide two new functional modules for curating the data that will be used as input to network inference procedures. The first module is devoted to handle missing data. If the toolbox detects that the raw dataset has missing values, the TSR method is applied in order to fill the holes in the dataset coherently with the latent structure of data. The second module detects extreme outliers in the raw dataset, and if there exist, they are first replaced by missing values and then recalculated using TSR. TSR was originally proposed in [14] for PCA model building with missing data, however, the application of TSR to correct outliers is new. The way both preprocessing modules act can be visualized in Fig. 1. To evaluate the performance of TSR a comparison with other missing data methods commonly used by practitioners is carried out using several network inference benchmark problems. Likewise, several univariate and multivariate outliers are included in the datasets in order to check the ability of the outlier detection module.

Additionally, to illustrate how a network inference method can be augmented with these functionalities, we use them here in combination with a state of the art technique called MIDER (Mutual Information Distance and Entropy Reduction) [19]. MIDER is a general purpose method that exploits information-theoretic concepts such as entropy and mutual information [20, 21]. Information-theoretic methods have strengths such as good scalability and the ability of detecting nonlinear relationships, and are widely used for the reverse-engineering of biological networks [22], with examples such as CLR [23], ARACNE [24], MRNET [25], MI3 [26], or TD-ARACNE [27]. In [19] the performance of MIDER was benchmarked against these methods, obtaining competitive results. We emphasize that MIDER is used for demonstration purposes, but any other network inference method – such as those mentioned before – could be used as well. The data curation modules presented here are of general purpose, and work on the data independently of the reverse engineering procedure.

To facilitate their use, a Matlab/Octave implementation of the two data curation modules is included in the Additional files section (Additional files 1 and 2). Additionally, they have been included in a new version of the MIDER toolbox, MIDERv2 (http://gingproc.iim.csic.es/~gingproc/mider.html and https://sites.google.com/site/midertoolbox/).

Network inference studies that take into account the missing data imputation problem have been more common in the social sciences than in the biological sciences; however, some examples of the latter type can also be found. Thus, the works [28–32] report network inference results obtained with datasets with missing values. Wu et al. [28] presented a network inference method with an interpolation controller, providing three selections of data interpolation approaches. In [29, 30] missing data is handled with a weighted *k*-nearest neighbor method. Hurley et al. [32] illustrated the use of a suite of GRN analysis tools imputing missing data using the LSImpute missing value estimation method. It should be noted that the aforementioned methods [28–30, 32] are specific for GRN inference with gene expression data, and the approaches chosen to handle missing data are not justified nor compared to other alternatives. In [31] a network inference tool designed for GRNs, NetGenerator, is extended in several ways in order to predict pathogen-host interactions (PHIs). Remarkably, NetGenerator is applicable to datasets with missing values, although it requires complete data for the last time point, which means that missing data imputation procedures may still be needed in some cases. There are also studies that have addressed the issue of missing data in biological applications, but outside the context of network inference, e.g. [33].

The present contribution differs from the aforementioned works in that it (i) presents a general method for handling missing data and outliers, (ii) compares its performance with that of other common approaches, (iii) provides an implementation of the methodology, and (iv) combines it with a freely available general-purpose network inference method. In this respect, there may be more resemblances with a recently published paper [34], which presents a framework for network inference in Cytoscape and includes the possibility of using three built-in missing data “apps”: row average, zero imputation, and Bayesian principal component analysis. The present manuscript is complementary to [34] since (i) the missing data imputation methods are different, and (ii) here, outlier detection and correction is also taken into account.

## Methods

This section begins with a description of the methodology for handling missing data, presenting the different methods used in the comparative study. Afterwards, the procedure for the detection and correction of outliers is defined. Then the main features of the MIDER network inference method are summarized, including an introduction of the concepts of entropy and mutual information, which are common to other information-theoretic methods. Finally, the case studies used for testing the methodology are described.

### Missing data

Two projection to latent structure methods are used in this paper for imputing missing data: the iterative algorithm (IA) and the trimmed scores regression (TSR). Since both use principal component analysis (PCA) in their cores, this method is introduced here. The aim of PCA is to find the subspace in the variable space where data vary the most [35]. The original variables, which are usually correlated, are linearly transformed into a lower number of uncorrelated variables (the so-called principal components, PCs). PCA follows the expression:

(1) $$ \textbf{X} = \textbf{T}_{A} \textbf{P}^{T}_{A} + \textbf{E}_{A}   $$

where **X** is the data matrix, *A* is the number of PCs extracted, **T**_*A*_ is the scores matrix containing the projection of the objects in the *A*-dimensional subspace, **P**_*A*_ is the loadings matrix containing the weights for the linear combination of the variables represented in each of the PCs, and **E**_*A*_ is the matrix of residuals.

Recently the TSR method [13] has been adapted to the PCA model building problem with missing data [14]. TSR was originally proposed for the PCA model exploitation problem, *i.e.* when a PCA model fitted on complete data is used to process incomplete new observations [13]. In our context, the model building version of the algorithm is applied.

Some notation has to be introduced to describe the TSR method. Let **x**_*i*_ be a row of the data matrix **X**. If this observation has missing values, the vector can be reordered to have all missing values, $\textbf {x}_{i}^{\#}$, at the beginning, and all known values at the end, $\textbf {x}_{i}^{*}$. This induces a partition on the original data matrix $\textbf {X}_{i}=\,[\!\textbf {X}_{i}^{\#} \textbf {X}_{i}^{*}]$, corresponding to the missing and known parts of row vector **x**_*i*_, respectively. Now the steps of TSR algorithm can be enumerated:

1. Impute 0s for all missing values.
2. Center the data subtracting the mean of all variables.
3. Fit a PCA model.
4. For each row *i* with missing data:
  - Fit a regression model between $\textbf {x}_{i}^{\#}$ and the scores of a new PCA model on $\textbf {x}_{i}^{*}$.
  - Estimate the missing values with the predictions of the regression model.
  - Incorporate the estimated missing values in the dataset **X**.
5. Add the mean of the original variables.
6. Repeat steps 2–5 until convergence, *i.e.* when the mean difference between the predicted values for the missing data in step *n* and *n-1* is lower than a specified threshold.

Another procedure for building a PCA model from an incomplete dataset **X** is IA [11]. This method differs from TSR in the imputation step. TSR fits a regression model between the missing values and the scores of the available data, while IA uses the PCA model calculated using all data. Since both TSR and IA use PCA internally, the number of PCs, *A*, have to be determined *a priori*. The choice of the number of PCs depends on the aim of the study [36, 37]. Here, since we want to carefully reconstruct the missing values, we need to capture a huge amount of variability in the PCA model. Therefore, *A* is here determined automatically, computing as many PCs as needed to explain 90 % of correlation in data.

There are other approaches to impute missing data that are commonly used by practitioners. One of these methods is the complete case analysis (CC) [9]. This method consists simply in the elimination of the rows in the dataset with missing values. Another typical approach is to impute the mean values of the variables with missing data, the so-called unconditional Mean Imputation (MI). These two methods are strongly discouraged by the authors, since they may involve a huge waste of data in the former case, and may destroy the correlation structure of the original data in the latter. However, since these methods are commonly used as a first (and fast) attempt to “solve” the problem of missing data, we decided to include here their results. Additionally, two other methods are included in the comparative study. The first one consists of imputing the average value of a linear interpolation (LI) between the previous and the posterior values of the missing datum. The second fills the missing values of an observation with the ones of its nearest neighbor (NN) using the *k*-nearest neighbors algorithm.

A key comment is due here regarding missing data pattern and mechanism [9, 38]. All aforementioned methods are applicable when the pattern, *i.e.* the positions in which the missing data appear in the data set, is nonstructured (the missing values are located in a random fashion in all rows and columns of the data set), univariate (missing data appear only in a single variable), or block-wise (the missing values appear only for a subset of variables). Additionally, the missing data mechanism, *i.e.* the reason why the missing values are missing, has to be missing completely at random (MCAR) or missing at random (MAR). When the missing value mechanism is not missing at random (NMAR) (*e.g.* questions not answered on purpuse in a survey or values below the detection limit of a device) these methods are not applicable, since there is not enough available information to infer the missing positions in the data. An extended discussion on these issues can be found in [9].

### Outlier detection and correction

As stated in the introduction, projection to latent structure methods are commonly used within industries to monitor huge amounts of variables during a process. The concept of abnormal situation (or fault) in this context can be extrapolated here to errors during the data acquisition or mistakes during the data compilation. With this objective, PCA can be applied to find the latent structure of the original data, before reconstructing the network, and then identify these uncommon measurements.

When a PCA model is fitted, two different types of outliers can appear [17]. The first type of outlier is detected via the Hotelling- *T* ^2^ statistic, which represents the estimated Mahalanobis distance from the center of the latent subspace to the projection of an observation onto this subspace [39]

(2) $$ T^{2} = \sum_{a=1}^{A}\frac{\textbf{t}^{2}_{a}}{\lambda_{a}}   $$

where *A* is the number of principal components (PCs) of the PCA model, the **t**s are the score vectors (columns of **T**_*A*_ matrix), and *λ* are the values in the (diagonal) covariance matrix of **T**_*A*_.

The second type of outlier is the Square Prediction Error (SPE), which measures the euclidean (perpendicular) distance from an observation to the *A*-dimensional latent subspace [39]:

(3) $$ SPE = \textbf{e}_{i}^{T} \textbf{e}_{i}   $$

where **e**_*i*_ is the *i*-th row of the residual matrix, **E**_*A*_, of the PCA model.

While the first type of outlier usually represents a change in the process, but still coherent with the actual correlation structure of the data, the second one breaks the correlation structure, which is usually associated to failures in the process. The latter type of outliers should be removed in order to better understand the true structure of data. This idea can be extrapolated to network inference, being the SPE outliers the possible failures when the dataset is built. According to the previous rationale, these outliers have to be removed in order to study the relationships between species; otherwise these anomalous values could mask the true structure of data. Once an observation is classified as an outlier, contribution plots can be used to isolate the original variable responsible of this abnormal behavior [40].

The present paper proposes a methodology for automatically detecting and correcting outliers. Some related approaches have been published in the past focusing on fitting PCA models with missing data and/or outliers [41]. Here, since the goal is to cure the dataset, we use TSR to correct for the outliers, thus allowing the exploitation of all the data available for the network inference task.

The outlier detection and correction procedure is as follows. The first step consists of calculating the PCA model of data. As in TSR, the number of PCs is here determined automatically, extracting all the PCs whose associated eigenvalue, in the singular value decomposition [42], is higher than one. It is worth noting that the number of PCs determined here may be different than the number of PCs extracted by TSR, since in the outlier scheme we want to detect deviations from the main directions of variability, *i.e.* deviations from the PCs with the highest eigenvalues.

Once the PCA model is fitted, the next step consists of calculating 95 % upper control limit of SPE in order to detect possible faults. Different ways of computing control limits have been proposed in the literature [40]. However, when dealing with real data, which do not fulfill the theoretical constraints, the outliers may not be correctly detected in this way. Hence in this study we calculate the control limits using a cross validation scheme [43] as follows: a thousand data subsets are randomly selected to compute “real” 95 % control limits, *i.e.* leaving 5 % of the observations above the limit. Then, the median of all the limits is computed to take it as the reference limit. Values above this limit are considered outliers.

From the set of outliers, another classification has to be done. Any control limit based on a confidence interval has intrinsically a false alarm rate, corresponding to the confidence level. In the present case, 5 % of the observations are likely to fall above the control limit without necessarily being outliers. If more observations appear, they can be considered as faults. To distinguish between both types of “outliers” we follow an outlier classification recently proposed in [44]. Firstly, the outliers are ordered in decreasing order. Secondly, the admissible false alarm rate, corresponding to 5 % of the observations, are no longer considered as outliers. Finally, each value above two times the control limit is considered an outlier. Additionally, a data point is considered an outlier if its distance to the control limit exceeds 10 times the distance between the lowest false alarm and the control limit.

Once the extreme outliers have been identified it is needed to determine the variable responsible for the fault. For this purpose, contribution plots can be used to determine which variable *k* of the *i*-th observation has the highest Square Prediction Error (SPE) [39]:

(4) $$ Cont(SPE,x_{i,k}) = e_{i,k}^{2}   $$

Once the responsible variable is identified, a missing value is generated for that value. And finally, TSR is again used to reconstruct the faulty observation following the latent structure of data.

### Network inference: using mutual information distance and entropy reduction (MIDER)

Here we briefly introduce some concepts relevant for information-theoretic network inference, which will be later needed for describing the MIDER method used to demonstrate our approach.

The theoretical foundation of network inference methods such as [19, 23, 25–27] is information theory, which is based on the concept of entropy as defined by Shannon [20]. The entropy of a discrete variable *X* with alphabet *χ* and probability mass function *p*(*x*) is:

(5) $$ H(X)=-\sum_{x\in \chi} p(x) \log p(x)  $$

where the logarithm to the base 2 is usually chosen. For continuous variables the $\sum $ is replaced by $\int $. It is possible to calculate the joint entropy of a pair of variables (*X*,*Y*) as $H(X,Y)=-\sum _{x}\sum _{y} p(x,y) \log p(x,y)$. Another important quantity, conditional entropy *H*(*X*|*Y*), can be calculated as the entropy of a random variable *Y* conditional to the knowledge of a second one, *X*:

(6) $$\begin{array}{@{}rcl@{}} H(Y|X) & = & \sum_{x} p(x) H(Y|X=x) \\&=&-\sum_{x}p(x)\sum_{y}p(y|x)\log p(y|x)= \\ &=&- \sum_{x}\sum_{y} p(x,y) \log p(y|x) \end{array} $$

The relation between joint and conditional entropy is expressed as *H*(*X*,*Y*)=*H*(*X*)+*H*(*Y*|*X*). A related concept is the relative entropy, which measures the distance between two distributions *p* and *q* and is defined as $D(p||q)=-\sum _{x} p(x)log \frac {p(x)}{q(x)}$. It has two important properties: it is always non-negative, and it is zero if, and only if, *p*=*q*. The relative entropy between the joint distribution *p*(*x*,*y*) and the product distribution of two variables, *p*(*x*)*p*(*y*), is called mutual information, *I*, that is:

(7) $$\begin{array}{@{}rcl@{}} I(X,Y)\!\!&=\!&\sum_{x}\sum_{y} p(x,y) \log \frac{p(x,y)}{p(x)p(y)}\,=\,H(X)\,-\,H(X|Y)= \\ \!&=&H(X)+H(Y)-H(X,Y) \end{array} $$

For a detailed description of the elements of information theory, see [21]. The concept of mutual information can be used to detect relationships between variables of any kind, since it is a measure of the amount of information that one random variable contains about another. Indeed, it has been used as the basis of many inference methods, *e.g.* the aforementioned [19, 23, 25–27], among others. Mutual information is a symmetric measure that does not assume any property of the dependence between variables, such as linearity or continuity. Hence it is more general than linear measures such as the correlation coefficient, and it has been shown that it is capable of inferring more interactions [23]. Intuitively, if two components of a network interact strongly, their mutual information will be large; if they are not related, their mutual information will be (theoretically) zero, or (in practice, when estimated from data) very small.

In this work we have built on the network inference method named MIDER [19], which uses information-theoretic concepts to infer relationships between variables, and to discriminate between direct and indirect interactions. Its core feature is entropy reduction, which consists of calculating the reduction of the entropy of a variable *Y* caused by other variables in the network. Theoretically, if a variable *Y* is completely independent of a set of variables **X**, then *H*(*Y*|**X**)=*H*(*Y*); otherwise *H*(*Y*|**X**)<*H*(*Y*). Therefore, if a subset of variables **X** reduces the entropy of *Y* to the minimum (*i.e.* if adding an additional variable to **X** does not produce further reductions in the entropy of *Y*), we have found the complete set of connections between *Y* and the remaining variables in the network.

The MIDER methodology uses the aforementioned concepts in the following procedure (for further details readers are referred to [19]):

1. Based on the estimation of time-lagged multidimensional entropies and mutual information, MIDER estimates the distance between variables and then projects the distance matrix onto a 2-D space using multidimensional scaling.
2. Then the links between variables are established based on the first, second and third order conditional entropies.
3. The strength of a link between two variables is calculated from the relative reduction of the entropy of the first variable caused by the addition of the second variable.
4. Finally, directionality is assigned to the inferred links. The direction of a link connecting two variables *X* and *Y* is the one that gives the maximum transfer entropy. The transfer entropy from *X* to *Y* is calculated as:
  (8) $$ T_{X \rightarrow Y}=H(Y^{t}|Y^{t-\tau})-H(Y^{t}|Y^{t-\tau}, X^{t-\tau})  $$
  where *t* indicates the lag - obtained in the first step - for the *X*−*Y* pair.

### Case studies

**Fig. 2:**
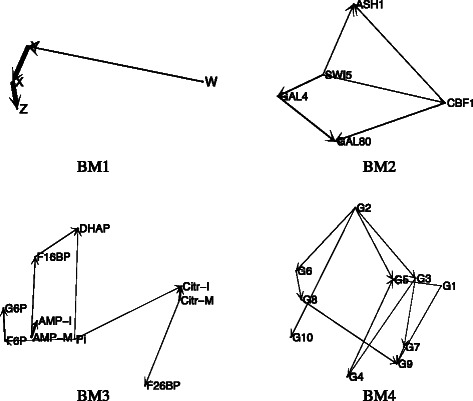
Case studies. MIDER reconstructed networks with the original data for benchmark problems BM1 (small chain of 4 reactions), BM2 (In vivo Reverse-engineering and Modeling Assessment), BM3 (first steps of a glycolytic pathway) and BM4 (DREAM4 in silico network challenge)

In order to validate the usefulness of the proposed methodology five well-known benchmark problems are selected, four from [19] and one new test case from the DREAM5 Network Inference challenge^1^. Additionally, to test the performance of the TSR method against the other approaches, a comparative study is also performed. The first benchmark problem (BM1) is a small chain of three reactions between four species (*W*, *Y*, *X* and *Z*) proposed in [45, 46] (see BM1 in Fig. 2). In this network the reaction between *W* and *Y* is much weaker than the other reactions *Y*−*X* and *X*−*Z*.

The second benchmark problem (BM2) is the so-called IRMA (In vivo Reverse-engineering and Modeling Assessment) [47]. It corresponds to a yeast synthetic network for benchmarking reverse-engineering approaches. IRMA consists of five genes that regulate each other through several interactions. It is particularly interesting as a benchmark because it is an engineered system, which means that the true network is known, and at the same time the system outputs can be measured in vivo, instead of just simulated in silico. A dataset consisting of time series and steady-state expression data after multiple perturbations is available; for network inference purposes the time-series data was used. Figure 2 shows the reconstruction of the network by MIDER.

BM3 models the first steps of a glycolytic pathway. The reconstruction of this network is shown in Fig. 2. The problem of reverse-engineering this system – a chemical reaction network of realistic size – was chosen in [48] as a way of demonstrating the feasibility of the Correlation Metric Method (CMC). With that aim, an experiment was carried out in a continuous-flow, stirred-tank reactor. Experimental time-series data were obtained for the concentrations of ten chemical species: Pi, G6P, F6P, F16BP, F26BP, and DHAP, as well as the input and reactor concentrations of citrate and AMP. The sampling period was 13 minutes, and the overall number of sampling instants was 57. The data is publicly available online^2^ as part of the Deduce software package.

The fourth benchmark problem (BM4) was generated for the DREAM4 in silico network challenge^3^. This challenge aimed at reverse engineering genetic networks. The artificial network presented here was generated as reported in [49, 50]. It consists of 10 nodes and 13 links. The MIDER reconstruction of this network is shown in Fig. 2.

Finally, the last benchmark problem (BM5) is an in-silico network produced for the DREAM5 network inference challenge. Specifically, we used the problem referred to as “Network 1”, which is an in silico network with 1643 nodes (genes), of which 195 are transcription factors. The challenge consisted in reporting an ordered list of 100000 predicted interactions. We used MIDER to establish this list from the conditional entropies, reproducing the original network with AUROC = 0.756, AUPR = 0.181. As a comparison, other information-theoretic methods used by the participants in the DREAM5 challenge reported solutions in the ranges AUROC = [0.625 - 0.773], AUPR = [0.106 - 0.255], showing that MIDER performed well when compared to other contenders. The reconstruction of the network is not shown here.

## Results and discussion

The performance of the two data preprocessing modules presented in this work has been assessed in two studies, whose results are reported in this section. The first one addresses the missing data imputation, in which a comparative study is performed among trimmed scores regression (TSR) and other methods commonly used by practitioners. The second one addresses the outlier detection, in which a set of outliers are simulated in case studies in order to be detected and corrected by the outlier module. A 2.9 GHz Intel Core i7 computer with 8 Gb RAM has been used to obtain all results in this section.

### Missing data: comparative study

In this section we show the results of the tests of the missing data module. The performance of the TSR method for the imputation of missing data is compared against another multivariate projection method, the iterative algorithm (IA), and other fast approaches used by practitioners, like the complete case analysis (CC), the mean imputation (MI), the linear interpolation (LI) between known time points and the nearest neighbor imputation (NN). The study is performed as follows. The five benchmark problems (BM1–BM5) described in the previous section are chosen as case studies. In BM1–BM4, 7 different percentages of missing data are generated, from 5 % to 35 %, and for each percentage, 100 datasets are simulated. For BM5 the same percentages of missing data were generated; however, only one dataset was simulated for each of them, due to the much higher computational cost of reverse engineering such a large-scale network compared to BM1–BM4.

In the original TSR paper [14], the mean square prediction error (MSPE) was used to assess the performance of each imputation method. However, in the present study the application is different: the aim is to use the imputed data for network inference. Hence, since different imputations may lead to the same inferred network, we use here instead the precision (*P*) and recall (*R*) of each method as the performance criteria to evaluate the results. *P* and *R* are defined as follows:

(9) $$ P=\frac{TP}{TP+FP} \quad \quad R=\frac{TP}{TP+FN}  $$

where *TP* are the true predicted links with respect to the reconstruction of the network without missing values, *FP* the false positives, and *FN* the false negatives.

**Fig. 3:**
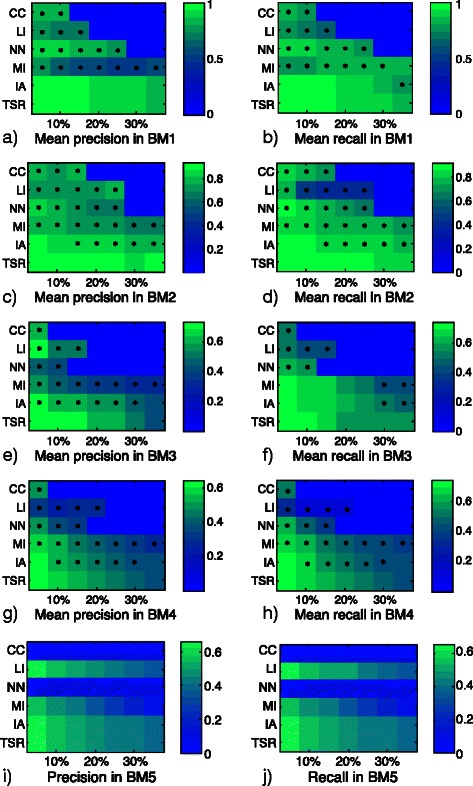
Results of the missing data comparative study. The results of the different methods are shown by rows: complete case analysis (CC), linear interpolation (LI), nearest neighbor algorithm (NN), mean imputation (MI), iterative algorithm (IA) and trimmed scores regression (TSR). By columns, the asterisks mark methods with a statistically worse performance, compared to TSR, in each percentage of missing values

The mean results of *P* and *R*, for each BM problem, are shown in color scale maps in Fig. 3. In each of the subplots, the rows correspond to the six imputation methods under study (CC, LI, NN, MI, IA and TSR) and the columns correspond to the percentages of missing values simulated on the complete dataset. Thus, each column permits to compare the results of TSR against the other approaches for a particular percentage of missing data. An absolute zero value, on either *P* or *R*, in Fig. 3 implies that the method is unable to impute the missing values for that particular percentage. To determine whether the differences in *P* and *R* obtained among TSR and the other methods, for each percentage, are significant or not, a mixed-effects three-way ANOVA is applied on the results. The first factor corresponds to the method, the second one to the percentage and the third one to the simulated dataset with missing values. Method and percentage are fixed-effects factors, while simulated dataset is a random-effects factor. The statistically significant differences between pairs are established using the 95 % LSD (least significance difference) intervals.

The asterisks on the color maps in Fig. 3 mark the statistically significant differences (p-values <0.05) with respect to TSR. In this way, if a method, with certain percentage, has an asterisk implies that TSR has significantly better results. When no symbols are placed on a method it implies that there are no statistically significant differences with respect to TSR.

Figure 3 shows that CC, LI and NN are not able to impute the missing data for medium-high percentages. CC is the worst method: for small networks (BM1 and BM2) CC can deal with up to 10–15 % of missing data, but for more complex networks (BM3 and BM4) it can only perform an imputation with 5 % of missing values. LI and NN are slightly better, but they fail to reach 30 % of missing data for all BMs. The TSR performance in all BMs is statistically better than CC, LI and NN. TSR results are superior to MI in most of the percentages of all BMs. Regarding IA, TSR attains statistically better results for most percentages in BMs 2–4. In the large network (BM5) no statistical differences can be computed, however, none of the aforementioned methods seems to outperform TSR in this case study. CC and NN are not applicable in BM5 since, even considering only 5 % of missing values, all rows in the data set have missing data. Furthermore, the results in this DREAM5 network are similar to the mean values obtained in DREAM4 network (BM4). Since no single method is statistically better than TSR for any percentage of any dataset, it is sensible to choose this method to implement the missing data module of network inference procedures.

The missing data imputation is achieved in around 1–2 seconds in BM1-BM4, for all methods; however, most of them are unable to impute with medium-high percentages of missing data. In BM5, LI maintains the computation time of BM1-BM4, the mean imputation (MI) is achieved in 48 seconds, and the most accurate methods in terms of *P* and *R* (IA and TSR) perform the imputation in 1 hour by truncating the number of PCs to 50 (to accelerate convergence).

Regarding the reconstruction of the network, TSR, when used in combination with MIDER, is able to recover more than 90 % of the links inferred with the complete dataset in small networks with low percentages of missing data. For higher percentages, 30–35 % of MD, it can infer 80 % of the links. Furthermore, for more complex networks with low percentages of MD, it is able to reconstruct nearly 70 % of the links inferred with complete data. When the % of MD increases in these networks, it can recover 40–50 % of the links.

### Outlier detection and correction: a simulated study

A simulated study is described here to assess the performance of the outlier detection and correction scheme. Two types of outliers are simulated on the benchmark problems: univariate and multivariate outliers. The first group involves outliers in the usual sense, *i.e.* values 3 times the interquartile range above (below) the 3rd (1st) quartile. The second group involves outliers that do not satisfy the aforementioned condition, *i.e.* they are not outliers in a univariate sense, but nonetheless alter the data correlation structure. This means that values above (below) the mean plus (minus) 1.5 times the standard deviation are moved to the other side of the mean value, *e.g.* if a variable has mean 0 and standard deviation 1, a value of 2 is moved to –2. This latter group of outliers requires a multivariate approach to be detected since they are not univariate outliers, *i.e.* they can not usually be detected using, for example, a box-whiskers plot.

Three percentages of outliers are simulated in each dataset: 1 % (with a minimum of 2 outliers), 5 % and 10 %. 100 rounds of univariate and multivariate outliers are simulated for each benchmark problem. A paired t-test with *α* Type-I risk =0.05 is used to determine if the solution provided by MIDER is significantly improved by the inclusion of the detection and correctionmodule.

Table 1 shows the results of the simulated study using univariate outliers. The precision (*P*) and recall (*R*) of the network reconstruction of MIDER and MIDER + outlier scheme are shown by rows. It is worth noting that the MIDER reconstruction of BM1 with univariate outliers has exactly the same links as MIDER with the original data set. However, the inclusion of the outlier detection and correction scheme presents no significant differences among the results. In BM2-BM3, the performance of MIDER + the outlier scheme is statistically superior to the results of MIDER on the faulty data. In this way, the network reconstructed correcting the detected outliers is more similar to the one inferred by MIDER using the original data. The results for BM4 are not statistically significant. Regarding BM5, no statistical differences can be computed among methods, since only one simulation per case is performed. However, the *P* and *R* results are coherent with the mean values obtained for BM4. The outlier detection and posterior imputation in BM1-BM4 is performed in 2–3 seconds. This procedure takes 2 minutes in the large network (BM5).

**Table 1:** Univariate outliers simulated study

Outliers	Method	BM1		BM2		BM3		BM4		BM5	
		P	R	P	R	P	R	P	R		
1 %	MIDER	100 %	100 %	84.4 %	89.7 %	89.3 %	85.22 %	92.0 %	91.3 %	99.9 %	99.9 %
	MIDER+OS	100 %	100 %	96.9 % ^∗^	97.0 % ^∗^	94.6 % ^∗^	91.3 % ^∗^	90.6 %	90.8 %	99.8 %	99.8 %
5 %	MIDER	100 %	100 %	85.1 %	87.7 %	90.7 %	84.6 %	88.7 %	88.3 %	99.8 %	99.8 %
	MIDER+OS	99.7 %	99.7 %	91.0 % ^∗^	92.3 % ^∗^	93.1 % ^∗^	88.1 % ^∗^	87.6 %	87.9 %	98.9 %	98.9 %
10 %	MIDER	100 %	100 %	85.7 %	88.0 %	86.9 %	79.8 %	84.4 %	86.4 %	99.4 %	99.5 %
	MIDER+OS	99.7 %	99.7 %	88.2 % ^∗^	89.7 % ^∗^	90.2 % ^∗^	81.8 % ^∗^	82.7 %	85.4 %	99.4 %	99.5 %

Precision and recall of the network inference of MIDER and MIDER + the outlier scheme (MIDER+OS)

The asterisks mark the statistically better results of MIDER+OS (p-value <0.05)

Table 2 shows the results of the simulated study using multivariate outliers. In this case, MIDER + the outlier scheme performed statistically better than MIDER in BM1-BM4 for all percentages of outliers. The differences are bigger when the network is simpler, *e.g.* BM1-BM2, however there is also a significant improvement in the quality of the solution in BM3-BM4 when MIDER is used with this module. Finally, as in the univariate outliers study, no statistical differences can be computed in BM5.

**Table 2:** Multivariate outliers simulated study

Outliers	Method	BM1		BM2		BM3		BM4		BM5	
		P	R	P	R	P	R	P	R	P	R
1 %	MIDER	66.7 %	66.7 %	84.9 %	88.2 %	86.9 %	82.0 %	88.3 %	88.8 %	99.9 %	99.9 %
	MIDER+OS	100 % ^∗^	100 % ^∗^	91.6 % ^∗^	94.6 % ^∗^	90.8 % ^∗^	88.6 % ^∗^	91.5 % ^∗^	91.2 % ^∗^	98.6 %	98.6 %
5 %	MIDER	65.7 %	65.7 %	83.7 %	86.8 %	83.0 %	74.7 %	79.6 %	83.1 %	98.6 %	98.6 %
	MIDER+OS	91.0 % ^∗^	91.0 % ^∗^	86.9 % ^∗^	90.5 % ^∗^	85.6 % ^∗^	80.2 % ^∗^	82.8 % ^∗^	85.1 % ^∗^	97.3 %	97.3 %
10 %	MIDER	64.0 %	64.0 %	80.6 %	83.2 %	70.1 %	69.7 %	70.4 %	77.3 %	98.8 %	98.8 %
	MIDER+OS	73.0 % ^∗^	73.0 % ^∗^	82.6 % ^∗^	85.8 % ^∗^	72.8 % ^∗^	71.1 % ^∗^	73.0 % ^∗^	79.2 % ^∗^	98.2 %	98.2 %

Precision and recall of the network inference of MIDER and MIDER + the outlier scheme (MIDER+OS)

The asterisks mark the statistically better results of MIDER+OS (p-value <0.05)

### Remark on computation times

The computational cost associated to these procedures is relatively modest, compared to that of performing network inference. For the benchmark problems used in this work, the cost of the TSR-based approach is of only a few seconds for networks of moderate size (BM1-BM4), while for the very large one (BM5, including thousands of genes) it is of two minutes for outlier detection and correction and one hour for missing data imputation. These values do not represent a significant increase in the computation times of the network inference procedure, which means that the proposed methodology is appropriate for problems of realistic size.

## Conclusions

This work has presented an enhancement of network inference methods consisting of two preprocessing modules for handling incomplete and faulty datasets. The first one is capable of imputing values for lost measurements coherently with the latent structure of data. The second module detects univariate and multivariate outliers and replaces the faulty measurements with new values coherently with the available data.

A comparison of different methodologies for handling missing data has led to two main conclusions. First, traditional approaches used by practitioners, like complete case analysis (CC), linear interpolation (LI), nearest neighbor algorithm (NN) and unconditional mean imputation (MI), have problems when the datasets have high percentages of missing values and when the complexity of the network increases. Second, since trimmed scores regression (TSR) performs significantly better than the previous methods in BM1-BM4, including the iterative algorithm (IA), it represents the best approach to deal with missing values in network inference.

Likewise, the module for detecting and correcting outliers proposed here also applies TSR for replacing faulty observations. The good performance of this approach has been shown by means of simulations with five benchmark problems. The results of the network inference in four benchmarks with simulated multivariate outliers are statistically better when the new module is used as a preprocessing step; results obtained with univariate outliers in the fourth benchmark are not statistically significant.

Due to the long computation time required to analyse the fifth benchmark, a single simulation is performed in this large-scale network. Therefore, no statistical differences are computed in this case study. However, the results of TSR in the comparative study and the outlier detection and correction are coherent with the results of BM4.

By extending MIDER with these new functionalities we have combined two different approaches for data analysis: information-theoretic and variance-based. The joint use of both methodologies increases significantly the number of datasets that can be used for network inference.

Crucially, both MIDER and the new preprocessing modules are general-purpose methods, which may be applied to networks of any kind – not only biological, but also from other areas of science – without requiring prior knowledge from the user. Furthermore, the missing data and outlier detection and correction modules can be used as a preprocessing step for other network inference methods.

### Software availability

Both data curation modules, trimmed scores regression (TSR) for missing data imputation and the outliers detection and correction scheme, have been included in a new version of the MIDER toolbox, MIDERv2 (http://gingproc.iim.csic.es/~gingproc/mider.html and https://sites.google.com/site/midertoolbox/).

## Endnotes

^1^ http://wiki.c2b2.columbia.edu/dream/index.php/D5c4.

^2^ http://genomics.lbl.gov/?pageid=44.

^3^ http://wiki.c2b2.columbia.edu/dream/index.php/D4c2.

## Additional files

Additional file 1
**TSR.** The source code of trimmed scores regression (TSR) algorithm for missing data imputation is provided ready to be used in MATLAB. (M 2 kb)

Additional file 2
**OUTLIERS.** The source code of the outlier scheme for detection and correction is provided ready to be used in MATLAB. (M 3 kb)
